# Dispositional optimism and open‐label placebo responses in hair cortisol concentrations and psychological distress—A randomized controlled trial

**DOI:** 10.1111/aphw.70217

**Published:** 2026-09-09

**Authors:** Carolin Liedtke, Michael Schaefer, Sören Enge

**Affiliations:** ^1^ Department of Psychology MSB Medical School Berlin Berlin Germany; ^2^ Institute of Neuroscience and Biopsychology for Clinical Application MSB Medical School Berlin Berlin Germany

**Keywords:** dispositional optimism, expectations, hair cortisol, open‐label placebos, psychological distress

## Abstract

Open‐label placebo (OLP) treatments show beneficial effects on various health‐related outcomes, but studies investigating OLP effects on physiological measures remain scarce. This randomized controlled trial examined the effect of a 4‐week OLP intervention on psychological distress and hair cortisol concentrations (HCC) in 202 healthy university students preparing for mandatory oral exams and whether dispositional optimism moderates the OLP effects. Participants were randomly assigned to an OLP or control group. Psychological distress was repeatedly assessed via negative affect, test anxiety, and subjective stress. HCC was measured before and within the intervention. Treatment expectations were additionally examined in interaction with optimism. Results show that OLPs significantly reduced psychological distress and HCC compared to the controls. Optimism moderated the OLP effect on HCC, with less optimistic individuals demonstrating the strongest reduction, independent of expectation. Optimism did not moderate OLP effects in psychological distress. However, the OLP effect on psychological distress depended on the three‐way interaction of group, optimism, and expectation. The results suggest that OLPs alleviate the psychophysiological impact of a real‐life stressor and indicate that optimism and expectation differently shape psychological and physiological OLP responses. These findings are discussed within the framework of the interactionist perspective.

## INTRODUCTION

Open‐label placebo (OLP) treatments—where individuals are transparently informed that they receive a placebo treatment—have shown beneficial effects in various self‐reported outcomes, including reduced pain intensity (Disley et al., 2021), cancer‐related fatigue (Zhou et al., 2018), and symptoms of psychological distress (Kleine‐Borgmann et al., 2021; Schaefer et al., 2019; Winkler et al., 2023), with small‐to‐medium‐sized effects in clinical and healthy samples (Spille et al., 2023; von Wernsdorff et al., 2021). However, in contrast to deceptive (conventional) placebos that have been shown to influence various physiological parameters (Meissner, 2014), studies investigating the OLP effects on objective outcomes such as physiological measures remain scarce and provide inconclusive findings (Fendel et al., 2025). There is evidence for changes in neurophysiological responses to OLP treatments using electroencephalographic (EEG) measures (Guevarra et al., 2020; Schienle et al., 2022), whereas findings for OLP effects on salivary cortisol, a stress‐related neuroendocrine marker, are currently limited and based on smaller samples (Olliges et al., 2022; Schaefer et al., 2021; Schneider et al., 2020). Since EEG measures and salivary cortisol primarily capture acute neurobiological responses to OLP administrations, potential long‐term psychophysiological effects of OLPs remain widely unknown. In naturalistic stress contexts, such as university exams, which are known to elicit strong cortisol responses through social‐evaluative threat and performance pressure (Preuß et al., 2010), OLP studies examining physiological stress markers are lacking.

In the realm of physiological stress research, the analysis of hair cortisol concentrations (HCC) is an innovative and non‐invasive method to retrospectively assess cumulative cortisol levels (Stalder et al., 2017). As a marker of the hypothalamus–pituitary–adrenal (HPA) axis activity, HCC reflects long‐term systemic cortisol secretion, providing a reliable and robust indicator of chronic or cumulative stress. Compared to salivary or plasma cortisol, HCC is less prone to situational fluctuations and captures cortisol levels incorporated into hair over several weeks or months (Stalder & Kirschbaum, 2012), making it particularly suited for longitudinal assessments. In addition to its established associations with academic stress (Stetler & Guinn, 2020) and stress‐related psychiatric symptoms (Stalder et al., 2017; Wosu et al., 2013), HCC has been shown to be a sensitive and validated biomarker for evaluating the effectiveness of stress management interventions (Gherardi‐Donato et al., 2023; Iglesias et al., 2015). Consequently, HCC might represent a promising parameter to determine whether OLP interventions exert long‐term physiological effects in real‐life stress contexts.

Several psychological mechanisms potentially explaining the effects of OLPs have been critically discussed. Primary theories include associative learning processes such as classical conditioning, as well as expectancy‐related mechanisms, while other perspectives emphasize non‐conscious processes such as predictive processing or embodied cognition (Buergler, Sezer, Gaab, & Locher, 2023; Colloca & Howick, 2018; Kaptchuk, 2018; Sandler et al., 2010; Schafer et al., 2015). Although these theories partially rely on those that apply to conventional placebo effects (e.g. Kirsch, 2018), studies suggest that the underlying mechanisms involved in OLPs may be different from deceptive placebos, particularly with respect to expectations (Ballou et al., 2022; Schaefer et al., 2022). OLP trials explicitly inform their participants to receive a treatment without any active ingredients, while deceptive placebo studies suggest the potential receipt of an active medication. The variability in deceptive placebo responses can be explained by different expectation levels (Flaten et al., 2006; Vase et al., 2005). Evidence regarding the role of expectations in OLP effects is emerging, although findings remain mixed so far (Barnes & Faasse, 2026; Buergler, Sezer, Gaab, & Locher, 2023; Schaefer et al., 2022, 2025). In OLP research, expectations are rather low (Pan et al., 2022), but providing a plausible rationale as expectation manipulation may nevertheless be essential for the occurrence of OLP effects (Locher et al., 2017). Furthermore, personality traits have been identified as relevant moderators of deceptive placebo effects, contributing to interindividual differences in the responsiveness and interacting with potential underlying mechanisms such as expectations (Jakšić et al., 2013). Whether and how personality traits moderate OLP responses, however, has not yet been systematically examined.

In conventional, deceptive placebo research, dispositional optimism has been most frequently examined among personality traits, showing relatively consistent associations with placebo responses (Geers et al., 2007, 2010; Kern et al., 2020; Morton et al., 2009). Dispositional optimism, conceptualized as a stable tendency to expect positive outcomes across various life situations, is associated with better physical health, adaptive coping with stressors, lower HCC levels, and better responses to psychiatric treatments (Coopersmith et al., 2024; Milam et al., 2014; Nes & Segerstrom, 2006; Zapater‐Fajarí et al., 2024). In deceptive placebo studies, optimism differently shapes placebo responsiveness depending on contextual factors (Darragh et al., 2014; Geers et al., 2005; Jakšić et al., 2013; Kern et al., 2020). For instance, *higher optimism* is associated with greater placebo responses in experimental pain paradigms (Geers et al., 2010; Morton et al., 2009), as demonstrated by lower subjective pain ratings in optimistic individuals. Conversely, in a psychosocial stress context, *lower optimism* predicted stronger placebo‐related decreases in subjective stress (Darragh et al., 2014), underscoring the impact of contextual factors on personality differences in placebo responding. In OLP research, only a few studies have examined the role of dispositional optimism yet. Zhou et al. (2018) found no substantial association between optimism and OLP effects on cancer‐related fatigue. Likewise, Locher et al. (2019) reported non‐significant effects of optimism on OLP responses in heat pain. Nonetheless, given the role of optimism in deceptive placebo responses, optimism may influence OLP effects in intervention contexts and outcome measures that are more closely aligned with the trait domain of optimism, such as stress contexts, where optimism has been shown to play a relevant role in stress responses and stress management (Jobin et al., 2014; Nes & Segerstrom, 2006).

Dispositional optimism modulates deceptive placebo responding through its interaction with situational expectations (Jakšić et al., 2013). Individuals high in optimism tend to show an attentional bias toward positive information and are more inclined to process positively framed messages (Geers et al., 2003; Isaacowitz, 2005). When positive placebo expectations are provided (e.g., ‘pills to improve sleep quality’), optimistic individuals benefit more from placebo treatments than in conditions without induced positive expectations (Geers et al., 2007). On the other hand, pessimistic individuals—characterized by doubts, worries about the future, and rather expecting the worst (Schueller & Seligman, 2008)—are more likely than optimists to follow negatively framed (nocebo) expectations under a deceptive placebo condition (Geers et al., 2005). Simultaneously, it has been demonstrated that less optimistic individuals may benefit more from affect‐related interventions, as they appear more sensitive to discrepancies between expected and experienced outcomes than highly optimistic individuals. Consequently, positively disconfirming experiences within the intervention may lead them to contrast away from their negative expectations, thereby enhancing positive affect (Geers & Lassiter, 2002; Predatu et al., 2024). To conclude, both higher and lower levels of optimism may promote placebo responses through different mechanisms, depending on their interaction with situational expectations. However, expectations and optimism might not universally determine placebo responses but only when such dispositional and situational factors are jointly relevant to the specific placebo effect (Geers et al., 2010; Hyland et al., 2007). To the best of our knowledge, no study has yet investigated the potential interaction between dispositional optimism and treatment expectations in shaping OLP responses.

To address these gaps, the present study investigated the effect of a 4‐week OLP intervention on HCC and psychological distress and examined whether dispositional optimism moderates the OLP responsiveness. As a real‐life stress scenario, we used mandatory oral university exams that need to be passed by the students, representing a powerful naturalistic stressor. Psychological distress was operationalized as a latent construct reflecting related but distinct manifestations of emotional strain during the exam preparation period, including negative affect, test anxiety, subjective stress, and exam‐related stress (e.g. Losiak & Losiak‐Pilch, 2020; Winkler et al., 2023). In our main assumption, we expect optimism to be associated with the OLP effect on both HCC and psychological distress, based on its associations with deceptive placebo responses, without predefining the direction of this relationship due to inconclusive findings in OLP research. Investigating optimism as moderator extends the currently limited evidence on personality traits as potential predictors of health‐related outcomes in the context of OLP interventions (Ballou et al., 2022; Locher et al., 2019; Zhou et al., 2018). Identifying such moderators could help explain interindividual differences in treatment responsiveness and may inform a more differentiated understanding of for whom OLP interventions are most effective. By assessing both physiological and subjective outcomes, the study also aimed to clarify whether potential OLP effects are confined to self‐reported experience or extend to psychophysiological stress responses, which has implications for the validity of OLP effects. As a secondary objective, we examined whether optimism and expectation interact in shaping OLP effects. This was intended to inform theoretical models of psychological mechanisms underlying OLP effects and perspectives that emphasize the interaction of dispositional and situational factors that influence placebo‐related responses (Geers et al., 2007).

## METHODS

### Sample

**FIGURE 1:**
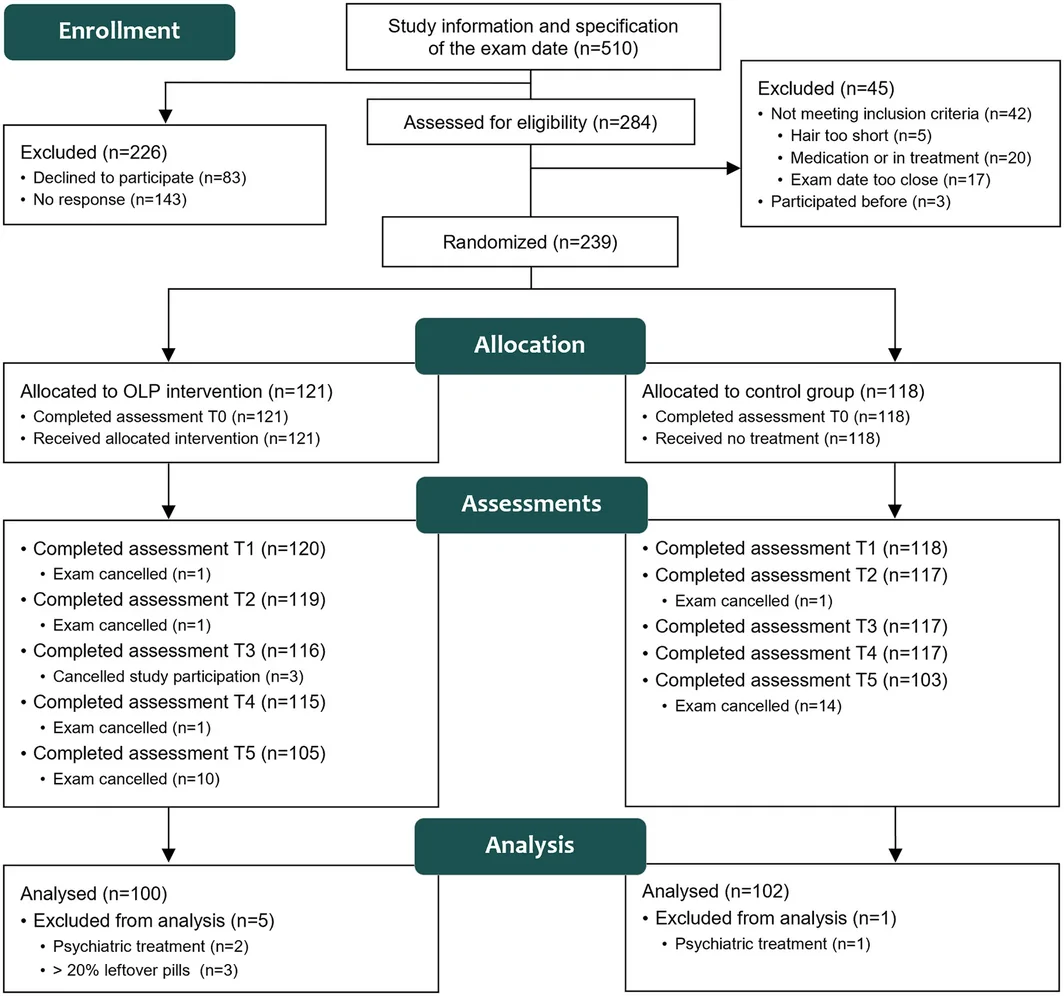
CONSORT flow diagram. The sample corresponds to that reported in Liedtke et al. (2026a).

The final sample included 202 healthy university students (mean age = 26.03 ± 4.53 years; 157 females) who were randomly assigned to either the OLP group (*n* = 100, 49.5%) or the control group (*n* = 102, 50.5%; see Figure 1 for flow diagram). Participants were recruited via university mailing lists and announcements during lectures. Eligibility criteria required students to have an upcoming oral exam and a minimum hair length of 3 cm. Exclusion criteria included a history of psychiatric, neurological, or endocrinological disorders or treatments, corticosteroid use, diabetes, allergies to sugar or artificial additives, heavy smoking (>10 cigarettes/day), regular use of illicit drugs, and pregnancy. Accordingly, three participants undergoing psychiatric treatment were excluded from analyses. In addition, participants in the OLP group with less than 80% adherence (*n* = 3) were excluded, following established clinical thresholds for good adherence (Simpson et al., 2006). Upon study completion, participants received 40 euros and six course credit hours. Demographic and baseline characteristics are summarized in Table 1.

**TABLE 1:** Demographic and baseline data for OLP and control group.

Characteristic	OLP group	Control group	*p*
*n*	100	102	
Females/males^a^	82/18	75/27	*p* = .148
Age (in years)	25.52 ± 4.10	26.53 ± 4.88	*p* = .113
T0 HCC (pg/mg)	4.17 ± 2.56	4.90 ± 3.50	*p* = .223
Negative affect (NA)	1.50 ± 0.50	1.44 ± 0.56	*p* = .463
Test anxiety (PAF)	45.30 ± 10.96	46.45 ± 11.63	*p* = .470
Subjective stress (DASS‐S)	6.26 ± 4.03	7.06 ± 4.56	*p* = .189
Exam‐related stress (VAS)	59.98 ± 29.92	65.22 ± 28.66	*p* = .205
Optimism	16.07 ± 4.23	16.92 ± 4.11	*p* = .148
Expectation	29.39 ± 17.79	30.62 ± 18.64	*p* = .633

*Note*: Mean ± 1 standard deviation and results from *t*‐test and 𝜒^2^ test are reported. HCC *n* = 176.

^a^ 𝜒^2^ test.

### Experimental design

In this randomized controlled trial (RCT), healthy university students provided written informed consent prior to participation and were then assigned to either an OLP intervention or a no‐treatment control group (analogous to Kleine‐Borgmann et al., 2021; Schaefer et al., 2019; Winkler et al., 2023). The study was conducted in accordance with the Declaration of Helsinki and registered in the Clinical Trials Register (DRKS00031423, 07/03/2023). It was part of a research project with multiple planned publications focusing on different research questions. Previous publications from this longitudinal RCT reported the psychophysiological effects of OLPs in a subsample (Liedtke et al., 2026b) and examined the moderating role of the five‐factor personality traits on OLP effects in the final sample, including all assessment waves (Liedtke et al., 2026a). The present article extends this work by investigating whether dispositional optimism and treatment expectations moderate psychological and physiological OLP responses in the final sample.

An a priori power analysis was conducted using linear multiple regression (fixed model, *R* ^2^ increase) in G*Power (Faul et al., 2009). Based on meta‐analytic evidence for OLP effects (Spille et al., 2023) and existing placebo studies reporting small‐to‐medium interaction effects involving individual‐difference variables (Ballou et al., 2022; Geers et al., 2007, 2010), the effect size for interactions was estimated at *f* ^2^ ≈ .05. With an *α* error probability of .05 and a desired power of .80, the required sample size was *N* = 160.

### Procedure and outcome measures

Baseline and demographic assessments were conducted 35 days before the exam (T0) and prior to randomization. Psychological distress was assessed using four self‐report measures: Negative affect (NA) was assessed with the 10‐item negative affect subscale of the German Positive and Negative Affect Schedule (PANAS; Breyer & Bluemke, 2016), which demonstrated good reliability in the present study (Cronbach's *α* = .85). Test anxiety was evaluated using the 20‐item German Test Anxiety Inventory (PAF; Hodapp et al., 2011), encompassing worry, emotionality, interference, and lack of confidence. The PAF showed high internal consistency (Cronbach's *α* = .91). Subjective stress was assessed via the 7‐item stress subscale (Cronbach's *α* = .84) of the German Depression Anxiety and Stress Scale 21 (DASS‐21; Nilges & Essau, 2021), and exam‐related stress was measured using a visual analogue scale (VAS, range 0–100; Klumbies et al., 2014), indicating how stressful the participants perceive the current situation in the context of the upcoming exam.

Dispositional optimism was measured using the German revised version of the Life Orientation Test (LOT‐R; Glaesmer et al., 2008). The test consists of 10 items, four of which are filler items that are not analyzed, whereas three items reflect the optimism subscale (1, 4, 10), and three items measure the pessimism subscale (3, 7, 9). The items are answered on a five‐point Likert scale from *strongly disagree* (0) to *strongly agree* (4). The overall optimism score is the calculated sum of the optimism and reversed pessimism subscales, with a good reliability (Cronbach's *α* = .80). After these assessments, all participants received written instruction explaining the placebo effect analogous to previous OLP studies (Kaptchuk et al., 2010; Schaefer et al., 2019) and watched a television news report about the open‐label placebo effect (analogous to Carvalho et al., 2016). Subsequently, treatment expectation was assessed using a VAS item (range 0–100), on which all participants indicated to what extent they expect the placebo pills to reduce their tension or anxiety (‘Estimate how much the placebo pills will reduce your tension/anxiety during the exam’).

Hair samples were collected at the baseline assessment (T0) and on exam day (T5). Two fine hair strands were obtained from the posterior vertex, and the 1‐cm segment nearest to the scalp was analyzed. Given that cortisol is continuously deposited in the growing hair shaft (Meyer & Novak, 2012) and human hair grows approximately 1 cm per month (Wennig, 2000), the 1‐cm segments reflect cortisol levels of the preceding month (T0 = pre‐intervention month, T5 = intervention month). Sample preparation and cortisol extraction were performed by an independent laboratory (Dresden LabService GmbH, Dresden, Germany) in accordance with the protocol described in Gao et al. (2013), using liquid chromatography–tandem mass spectrometry (LC–MS/MS), a highly sensitive method for steroid quantification. Intra‐assay coefficients of variance ranged from 3.8% to 12%, and the limit of quantification was 0.3 pg/mg.

Following these baseline assessments, randomization to the OLP or control group was performed via sealed opaque envelopes, with 1:1 allocation ensured by blinded research assistants. The OLP group received 56 placebo pills to take twice daily for 4 weeks, while the control group received no intervention but was informed about the option to obtain post‐trial placebo pills.

Over the following 4‐week intervention, psychological distress was repeatedly assessed via online questionnaires at 28 (T1), 21 (T2), 14 (T3), and 7 days (T4) before the exam. On exam day (T5), participants completed self‐report measures immediately before and after the oral exam and provided the second hair sample. OLP group participants were asked to return remaining placebo pills to measure adherence; control group participants indicated whether they were disappointed with their group assignment.

### Statistical analysis

#### Data preprocessing

HCC values were log‐transformed to correct positive skewness (Enge et al., 2020), and extreme baseline values were excluded using the 2.5th and 97.5th percentiles as cutoffs analogous to previous studies on steroid hormones (Bae et al., 2019; Okutan et al., 2024; Ueland et al., 2022), resulting in a final HCC sample of *n* = 176 (OLP *n* = 85, control *n* = 91) due to 10 outliers, as well as missing cases in HCC (*n* = 1) and relevant covariates (*n* = 15). Next, a change score was calculated by subtracting the baseline (T0) from the intervention‐month value (T5). For each self‐report measure (NA, PAF, DASS‐S, VAS), the area under the curve with respect to increase (AUCi) was computed to summarize within‐intervention changes from T1 to T5. Next, employing the lavaan package in RStudio (v4.3.2), a confirmatory factor analysis (CFA) modeled psychological distress as a latent factor based on the four AUCi indicators, capturing the common variance across conceptually related indicators and therefore the underlying psychological distress construct. The CFA was conducted to evaluate a theoretically driven measurement model of psychological distress. NA AUCi served as the reference indicator (loading fixed to 1). One extreme outlier was excluded, and robust maximum likelihood estimation (MLR) was used due to non‐normality. Model fit was evaluated using standard indices and their established cutoffs (Comparative Fit Index [CFI]/Tucker‐Lewis Index [TLI] ≥ .95, Root Mean Square Error of Approximation [RMSEA] ≤ .06, Standardized Root Mean Square Residual [SRMR] ≤ .08, and χ^2^) (Hu & Bentler, 1999). The resulting latent factor was extracted for main analyses.

#### Data analyses

Baseline and demographic values were examined using independent *t*‐tests or *χ* ^2^ tests to ensure that there were no group differences prior to the intervention. To test our main assumption, we conducted simple moderated regression analyses examining the influence of optimism on OLP responses, with psychological distress or HCC as dependent variables, group as independent variable (0 = OLP, 1 = control), and mean‐centered optimism as the moderator. Main effects of group and interaction effects between group and optimism were reported. In a secondary analysis, moderated regression analyses examined the three‐way interaction of group, optimism, and expectation on HCC and psychological distress. Simple slope analyses were reported in case of a significant interaction. Bootstrapping with 5000 resamples and heteroscedasticity‐consistent standard errors (HC3) was used to compute the 95% confidence interval (CI).

Age, sex, and job hours were included in all analyses as a priori defined covariates with respect to their previously reported associations with HCC and psychological distress (Drapeau et al., 2014; Mounsey et al., 2013; Thomsen et al., 2005; Wosu et al., 2013) as well as our study design (due to time constraints during exam preparation for working students) and sample composition. As the semester, degree program, and significant pre‐intervention stressors were significantly associated with the psychological distress measures, these variables were additionally controlled in the analyses examining psychological distress. In addition, baseline psychological distress was included as covariate, given its significant associations with optimism (see Table S1 for correlations and Methods S1 for latent factor modeling of baseline distress). HCC analyses were controlled for physical activity, BMI, UV exposure, hair washing frequency, sample weight, hormonal contraceptives, season, and baseline HCC, which were a priori defined confounders of HCC based on previous findings and recommendations (Frisch et al., 2020; Staufenbiel et al., 2015; Wosu et al., 2013). Statistical analyses were performed using IBM SPSS Statistics 27.0, with two‐tailed tests and a significance level of *α* = .05.

## RESULTS

### Sample characteristics and latent factor modeling

The baseline values and demographic characteristics showed no significant group differences before the OLP intervention (all *p* > .05; see Table 1). Prior to conducting the CFA, we examined the intercorrelations among the four AUCi variables. These were positive and statistically significant, indicating modest‐to‐moderate shared variance across measures (Pearson *r* = .269 to .454, all *p* < .001; see Table S2 for correlation matrix). The CFA modeling the latent factor psychological distress showed acceptable global fit indices, *χ* ^2^(2) = .222, *p* = .895; robust CFI = 1.000; robust TLI = 1.047; robust RMSEA = .000 (90% CI [.000, .068]); SRMR = .007. These fit indices may be biased in simple models with few degrees of freedom (e.g. Brown, 2015; Shi et al., 2021) and should therefore be interpreted with caution, considering additional model information. The standardized factor loadings indicated that the observed indicators (AUCi variables) loaded moderately to strongly on the latent factor of psychological distress (λ = .52 to .83, *p* < .001), and the reliability of the latent factor was acceptable (McDonald's ω = .71). Overall, these results suggest that while the indicators reflect conceptually distinct facets of psychological distress, they share sufficient common variance to justify a parsimonious one‐factor representation (see Table S3 for comparison with alternative models). Factor scores representing this shared variance were used in subsequent analyses.

### OLP effects and the moderating role of optimism

**FIGURE 2:**
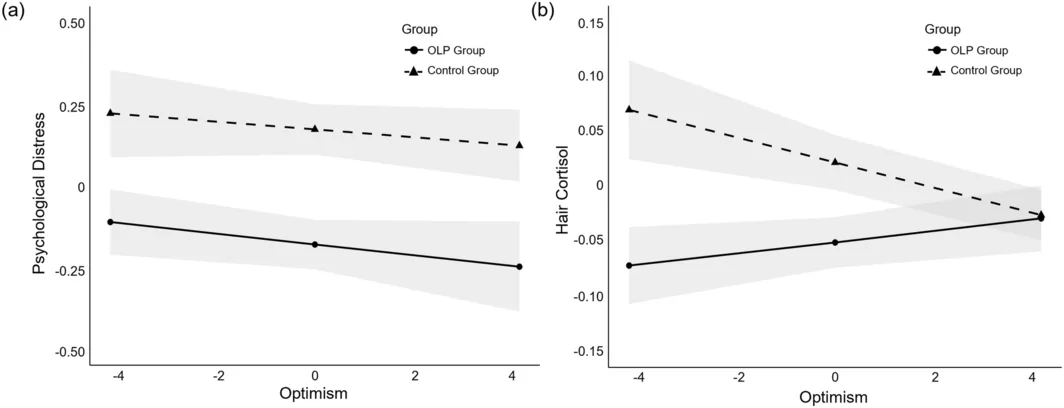
Simple slope plots of moderated regression analyses showing the association between dispositional optimism and (a) psychological distress (*n* = 201) and (b) hair cortisol (*n* = 176) for OLP and control group. Shaded areas represent ±1 standard error around the regression lines.

The simple moderated regression analysis revealed a significant overall model, *F*(10,190) = 3.313, *p* = .001, *R* ^2^ = .114, and a significant group effect on psychological distress, *b* = .352, *SE* = .114, *t*(190) = 3.104, *p* = .002, 95% CI [.129, .576], *ß* = .241, demonstrating lower psychological distress in the OLP group compared to the control group (see Figure S1 for the trajectories of each psychological distress measure). The interaction between group and optimism was not significant, Δ*R* ^2^ < .001, *b* = .005, *SE* = .029, *t*(190) = .161, *p* = .872, 95% CI [−.052, .061], *ß* = .019, *f* ^2^ = .0003, indicating that the group effect in psychological distress is not substantially moderated by optimism (see Figure 2a).

The simple moderation model with HCC as the dependent variable was significant, *F*(15,160) = 4.808, *p* < .001, *R* ^2^ = .284, and showed significant group differences, *b* = .073, *SE* = .035, *t*(160) = 2.100, *p* = .037, 95% CI [.004, .141], *ß* = .153, with lower HCC in the OLP group. The interaction between group and optimism was significant (see Figure 2b) and accounted for additional explained variance of 2.0% in HCC, *b* = −.017, *SE* = .008, *t*(160) = −2.051, *p* = .042, 95% CI [−.032, −.001], *ß* = −.199, with a small effect size (*f* ^2^ = .028). The simple slope analysis showed that the group effect on HCC was significant at low levels (−1 SD: *b* = .142, *SE* = .057, *p* = .014, 95% CI [.030, .255]) and average levels of optimism (*b* = .073, *SE* = .035, *p* = .037, 95% CI [.004, .141]) but not at high levels of optimism (+1 SD; *b* = .003, *SE* = .038, *p* = .945, 95% CI [−.072, .078]; see Table S4 for conditional estimated means).

Correlation analyses showed no significant relationship between changes in HCC and psychological distress in the total sample (*r* = −.014, *p* = .850), and within the groups (OLP group: *r* = −.104, *p* = .323; control group: *r* = .031, *p* = .764). In the control group, disappointment about group assignment was not significantly correlated to psychological distress (*r* = −.078, *p* = .436) and HCC (*r* = .086, *p* = .433).

### Interaction between optimism and expectations in predicting OLP effects

**FIGURE 3:**
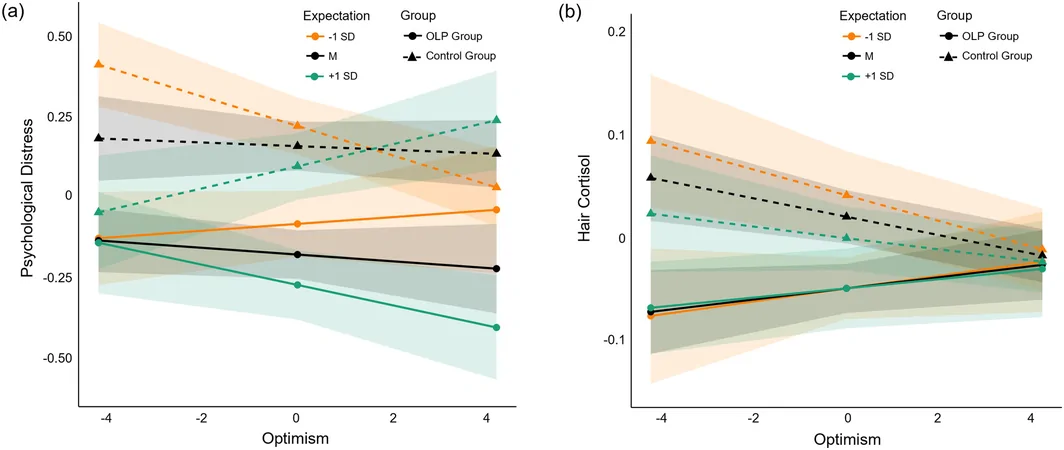
Simple slope plots of moderated regression analyses showing the three‐way interactions between dispositional optimism, expectation, and (a) psychological distress (*n* = 201) and (b) hair cortisol (*n* = 176) for OLP and control group.

Dispositional optimism and treatment expectations were not significantly correlated (*r* = .037, *p* = .603) in the total sample. The overall moderated regression model testing the three‐way interaction of group, optimism, and expectations in predicting psychological distress was significant, *F*(14,186) = 3.494, *p* < .001, *R* ^2^ = .166. The three‐way interaction was also significant, *b* = .003, *SE* = .001, *t*(186) = 2.321, *p* = .021, 95% CI [.001, .006], *ß* = .292, showing a small effect size (*f* ^2^ = .036) and explaining an additional 2.9% of the variance in group effects on psychological distress (see Figure 3a). Simple slope analyses revealed that at lower levels of optimism (−1 SD), the group effect was significant at lower levels of expectations (*b* = .543, *SE* = .199, *p* = .007, 95% CI [.151, .935]), approached significance at moderate expectations (*b* = .319, *SE* = .164, *p* = .053, 95% CI [−.005, .642]) but not at high expectations (*b* = .095, *SE* = .236, *p* = .688, 95% CI [−.371, .561]). Conversely, higher optimism levels (+1 SD) predicted group effects at higher expectations (*b* = .648, *SE* = .219, *p* = .004, 95% CI [.215, 1.080]) and less pronounced at moderate expectations (*b* = .359, *SE* = .165, *p* = .031, 95% CI [.034, .684]) but not at lower expectations (*b* = .070, *SE* = .223, *p* = .754, 95% CI [−.370, .510]), suggesting that the group effect on psychological distress depends on the interaction between optimism and expectation, with the strongest OLP effect when optimism and expectations are either both low or both high. At average levels of optimism, the group effect was significant regardless of expectation levels (low: *b* = .306, *SE* = .140, *p* = .029, 95% CI [.031, .582]; mean: *b* = .339, *SE* = .112, *p* = .003, 95% CI [.117, .561]; high: *b* = .371, *SE* = .155, *p* = .017, 95% CI [.066, .676]), suggesting that the OLP effect in psychological distress emerges in average optimism independently of expectation level (results remained comparable when baseline distress was residualized from the AUCi values instead of being included as a covariate in the moderation model).

The overall moderated regression model with the three‐way interaction explaining HCC was significant, *F*(19,156) = 3.679, *p* < .001, *R* ^2^ = .289. The three‐way interaction between group, optimism, and expectation was not significant, Δ*R* ^2^ = .001, *b* = .0002, *SE* = .0005, *t*(156) = .525, *p* = .600, 95% CI [−.001, .001], *ß* = .058, *f* ^2^ = .002, indicating that the effect of optimism on group differences in HCC did not vary depending on participants' expectations (see Figure 3b and Tables S5 and S6 for conditional estimated means).

## DISCUSSION

The present study examined the effect of a 4‐week OLP intervention on HCC and psychological distress in a naturalistic stress context—oral university exams that need to be passed—and explored whether optimism moderated the OLP effects. The OLP intervention significantly reduced psychological distress in university students during the 4 weeks until the exam compared to the control condition. This finding aligns with prior research showing that non‐deceptive placebos can diminish self‐reported psychological distress (Kleine‐Borgmann et al., 2021; Schaefer et al., 2019; Winkler et al., 2023). Most importantly, we demonstrated that OLP effects can be sustained across a 4‐week period using a powerful real‐life stressor, thereby extending these previous findings. The prevalence of psychological distress among university students is substantially higher than in the general population, with distress being linked to problematic health behaviors and impaired academic performance (Sharp & Theiler, 2018; Stallman, 2010), highlighting the need for sustainable, low‐risk stress management interventions. Perceived stress and distress symptoms typically increase during exam periods (Fritz et al., 2021), with highly stressed students more frequently resorting to neuroenhancing substances, potentially leading to adverse side effects (Maier et al., 2013). Within this framework, our findings suggest the potential utility of OLP treatments as an effective intervention to mitigate the health‐related impact of prolonged stress exposure in vulnerable populations.

Beyond subjective measures, the OLP group further exhibits significantly lower HCC levels compared to the control group. As a neuroendocrine marker of long‐term HPA axis activity and chronic stress (Stalder et al., 2017), HCC has not been previously investigated in placebo research before the present research project. In view of other indicators of HPA axis response, our finding corresponds with evidence demonstrating conventional placebo effects on acute salivary and plasma cortisol levels (Balodis et al., 2011; Peciña et al., 2013) and extends previous OLP studies that found no overall effect on salivary cortisol (Olliges et al., 2022; Schaefer et al., 2021; Schneider et al., 2020). While short‐term elevated cortisol levels may also imply adaptive responses to stressors, long‐term cortisol elevation is assumed to have maladaptive effects, leading to physical and mental health risks (Russell & Lightman, 2019). Among university students, HCC levels are substantially higher during the academic term compared to the semester break (Stetler & Guinn, 2020). Throughout the academic term, the exposure to stressful events such as performance demands or social‐evaluative threat is positively associated with HCC, emphasizing the association between academic stressors and HCC. Intriguingly, the present OLP intervention reduced HCC during exam periods, indicating potential benefits of a non‐deceptive placebo administration even on stress‐related neuroendocrine processes. HCC and psychological distress were not significantly associated, aligning with prior evidence suggesting weak or inconsistent associations between subjective and physiological stress measures (Campbell & Ehlert, 2012; Stalder et al., 2017). This pattern likely reflects that HCC indexes cumulative cortisol secretion over weeks, whereas self‐reported distress captures more proximal and transient experiences, indicating that both measures assess related but (temporally) distinct aspects of stress responding (Weckesser et al., 2019). Thus, our results support the evidence that HCC provides a suitable biomarker to evaluate stress‐related interventions (Gherardi‐Donato et al., 2023; Iglesias et al., 2015). The present findings build on our previous work from the same longitudinal RCT, in which OLP‐related reductions in psychological distress and HCC were first reported in a subsample (Liedtke et al., 2026b) and later examined in relation to the five‐factor personality traits in the final sample (Liedtke et al., 2026a). Here, we replicate the previously reported psychophysiological OLP effects and extend these findings by investigating them in relation to dispositional optimism and treatment expectations.

In line with our main assumption, dispositional optimism moderated the OLP effect on HCC. Specifically, individuals with lower optimism showed the strongest response to the OLP intervention as reflected in reduced HCC, whereas at increasing levels of optimism, group differences diminished. Optimistic individuals show flexible, effective coping with stressors (Nes & Segerstrom, 2006), exhibit lower HCC levels (Milam et al., 2014; Zapater‐Fajarí et al., 2024), and have reduced diurnal salivary cortisol profiles, even when their perceived stress increases (Jobin et al., 2014). It is therefore assumed that optimism buffers against stress, translating into adaptive physiological and psychological stress responses. Given this, group differences in HCC in the present study did not emerge among individuals high in optimism, potentially due to their higher stress resilience and generally lower cumulative cortisol levels, thereby leaving limited margin for further reductions based on the OLP intervention. This also aligns with the resistance hypothesis, indicating that individuals with higher levels of a specific disposition may be less responsive to interventions targeting outcomes that are already favorably influenced by their stable traits (McCullough et al., 2004).

Individuals low in optimism tend to expect negative outcomes in future events and to doubt their ability to successfully achieve their goals, enhancing the likelihood of anticipated failure (Schueller & Seligman, 2008). In response to stress, less optimistic individuals are more likely to engage in maladaptive coping, such as emotion avoidance, denial, or withdrawal, rather than employing active problem‐solving approaches, suggesting a poorer psychological adjustment to stressors (Nes & Segerstrom, 2006). This is also reflected in their physiological stress response, as high levels of perceived stress are associated with elevated salivary cortisol levels among individuals with low optimism (Jobin et al., 2014). Additionally, lower optimism scores are linked to slower cortisol and cardiovascular recovery after acute psychological stress (Brydon et al., 2009; Puig‐Perez et al., 2015). This stronger and more prolonged psychophysiological reactivity might contribute to increased cumulative cortisol secretion, which is reflected in higher HCC levels (Milam et al., 2014; Zapater‐Fajarí et al., 2024). In line with this, the elevated HCC levels observed among less optimistic individuals in the control group may reflect heightened long‐term HPA axis response, potentially due to their reduced stress resilience.

On the other hand, their increased stress vulnerability appears to make them more responsive to the OLP intervention, as reflected in reduced HCC in the OLP group that is particularly pronounced in less optimistic individuals. This finding suggests that OLPs may diminish less optimistic individuals' typically stronger prolonged physiological stress response. This is the first evidence that indicates a moderating role of optimism on OLP effects (Locher et al., 2019; Zhou et al., 2018), with an effect pattern that contrasts several findings from deceptive placebo research demonstrating that rather high optimism is associated with increased placebo responses (Geers et al., 2010; Kern et al., 2020; Morton et al., 2009). This divergence suggests that optimism may differently influence placebo effects depending on certain contextual conditions, aligning with previous investigations (Darragh et al., 2014; Geers et al., 2005). Indeed, within a psychosocial stress context, relatively comparable to the present study context, Darragh et al. (2014) similarly reported stronger placebo effects among individuals low in optimism. Given that researchers previously hypothesized that OLPs could lead to more adaptive coping in highly stressed individuals (Winkler et al., 2023), one may argue that OLPs could serve as a resource for external coping that compensates for less optimistic individuals' ineffective stress coping strategies, thereby supporting more adaptive HPA axis functioning (Janson & Rohleder, 2017). In addition, the tendency to stronger psychophysiological stress reactivity might leave greater space for improvements, making individuals with lower optimism potentially more sensitive to OLP intervention‐related changes in HCC.

In a secondary analysis we further examined whether treatment expectations interact with optimism's influence on OLP effects, since expectations are critically discussed as potential mechanism for OLP response (Buergler, Sezer, Gaab, & Locher, 2023; Locher et al., 2017; Schaefer et al., 2022). The influence of optimism on HCC was independent of treatment expectations, indicated by the non‐significant three‐way interaction between group, optimism, and expectation. This finding implies that HCC as a long‐term physiological measure may be more strongly influenced by stable dispositional tendencies—such as optimism—than by situational expectations. In line with that, it has been shown that HCC is rather less influenced by state‐related factors (Stalder et al., 2012). Placebo effects that are affected by optimism‐expectation interactions indeed were manifested in short‐term, state‐related self‐reports (Geers et al., 2005, 2007), whereby it remains unclear whether those placebo responses translate into neuroendocrine effects. Thus, OLPs may counteract heightened HCC levels associated with lower optimism independent of the interaction with treatment expectation, highlighting their potential to support adaptive physiological stress responses in vulnerable individuals.

The moderation of optimism on group differences in psychological distress was not significant, suggesting that optimism may differently shape subjective versus physiological placebo responses, as also indicated by prior investigations (Darragh et al., 2014; Geers et al., 2007; Peciña et al., 2013). Compared to the cumulative cortisol levels in hair, self‐reported states may be more volatile and sensitive to situational influences. According to the interactionist perspective by Geers and colleagues (Geers et al., 2005, 2007), situational factors (such as anticipated symptom improvements) may determine whether and how personality traits shape placebo responding in affective experiences. It is therefore conceivable that optimism affects OLP responses on self‐reported state measures primarily in interaction with situational factors and not independently, as previously demonstrated in deceptive placebo studies (Geers et al., 2007, 2010; Morton et al., 2009). Indeed, a significant three‐way interaction with group, optimism, and treatment expectation suggests that the group differences in psychological distress varied depending on both participants' dispositional optimism and their situational treatment expectations, aligning with the interactionist approach mentioned above (Geers et al., 2005, 2007). Interestingly, optimism and expectation were not significantly associated in the present sample, further suggesting that they represent empirically distinct constructs (dispositional vs. situational factors) that may, however, interact in shaping placebo responses. Thus, conscious treatment expectations appear to play a more relevant role in optimism's influence on subjective OLP effects than on the physiological effect in HCC. Intriguingly, the interaction effect is most pronounced when optimism and expectations are aligned, with stronger OLP responses observed when optimism and expectations are either both low or both high.

The finding of stronger OLP effects among highly optimistic individuals with high treatment expectations is consistent with previous evidence. Optimists particularly profit from interventions when positive expectations are experimentally induced, whereas negative or ambiguous expectations lead to non‐significant intervention‐related changes in affective experiences (Geers & Lassiter, 2002; Predatu et al., 2024). Although we did not intentionally induce different expectations but instead measured participants' individual treatment expectations, the observed effect pattern corresponds to studies in which expectations were deliberately manipulated (Geers et al., 2007; Geers & Lassiter, 2002; Predatu et al., 2024). Optimistic individuals are less sensitive to inconsistencies (negative expectation vs. positive experience) and assimilate their affective response to their prior expectation regardless of the actual experience (Geers & Lassiter, 2002). This tendency may explain why high expectations contributed to stronger OLP responses in psychological distress among highly optimistic individuals, whereas lower expectations led to non‐substantial OLP effects, representing the same assimilative mechanism.

Simultaneously, individuals with low optimism and low expectations exhibit stronger OLP responses in psychological distress. As less optimistic individuals are generally more sensitive to discrepant information, they tend to contrast their affective reactions when experience and expectation diverge (Geers & Lassiter, 2002). That is, when their negative expectation does not concur with a positive experience, they contrast the affective response away from the expectation, potentially enhancing favorable outcomes. For instance, low‐optimists with a negative expectation for positive experiences benefited more from these experiences than high‐optimists with the same expectation (Geers & Lassiter, 2002; Predatu et al., 2024). The beneficial effect of the OLP intervention on psychological distress thus may have been not consistent with the low‐optimistic individuals' low expectations, leading them to contrast their emotional experience.

In contrast, individuals low in optimism with high expectations did not exhibit significant OLP responses in the current study. However, research on this effect pattern is more inconsistent. Predatu et al. (2024) reported that less optimistic participants with positive expectations still benefit from a brief gratitude intervention, whereas Geers et al. (2007) found that low optimists in a positive expectation condition tended to profit less from a placebo treatment to improve sleep quality. One may speculate that participants low in optimism with high expectations may have held unrealistic anticipations regarding the OLP treatment, given their generally higher stress vulnerability (Jobin et al., 2014). Unrealistic treatment expectations may predispose individuals to become quickly discouraged, potentially leading to worse outcomes (Mancuso et al., 2001), which might explain the absence of the OLP effect among lower optimists with high expectations.

Several limitations and implications should be acknowledged. The sample intentionally comprised healthy university students to examine the OLP effect within controlled and comparable exams as stressors, which, however, restrict generalizability to other populations who may differ in age, health status, and educational background. While the sample size is relatively large compared to other OLP studies, replication in other cohorts and study contexts would be desirable to further establish the generalizability of these findings. Despite being sufficiently powered to detect effects of the anticipated magnitude, the present study may have limited sensitivity for identifying rather small higher order interaction effects, which require more power. The inclusion of multiple covariates, although primarily selected a priori based on theoretical and empirical considerations, may have reduced statistical power to detect smaller effects while improving the precision of the estimated effects by reducing residual variance. Although the study was not preregistered, the objectives were derived a priori from existing theories and empirical findings; nevertheless, future preregistered replication studies are warranted to strengthen the robustness of the findings. Furthermore, the transparent administration of placebos may introduce response biases in self‐reported measures. However, the observed OLP effect in HCC supports the validity of our findings in psychological distress. In the control group, 39.2% of the participants reported being disappointed about their group assignment, which is within the expected range reported in previous OLP studies (Buergler, Sezer, Bagge, et al., 2023; Schaefer & Enge, 2024). Previous OLP studies have discussed disappointment about group assignment as a potential source of bias that may influence group differences (Buergler, Sezer, Bagge, et al., 2023; Kleine‐Borgmann et al., 2021; Schaefer & Enge, 2024). However, in the present study, disappointment was not significantly correlated to psychological distress and HCC, suggesting that it is less likely to explain the OLP effects. Following procedures used in previous clinical trials to minimize potential disappointment effects (Carvalho et al., 2016; Kleine‐Borgmann et al., 2019), the control participants were offered the opportunity to obtain OLP pills after their study participation. Nevertheless, future studies should continue to employ designs that further minimize allocation‐related biases. Although beyond the scope of the present study, future research using multilevel models could examine the temporal dynamics of OLP effects and whether individual differences influence the timing and shape of OLP‐related changes. Moreover, it remains unclear how the OLP instruction shapes expectations, as it does not intentionally induce clearly positive or deceptive expectations. Future studies should clarify this to become a more detailed understanding of the role of expectation in OLP treatments. As the direct association between expectation or optimism and the OLP effect remains inconclusive in previous studies (Guevarra et al., 2020; Locher et al., 2019; Schaefer et al., 2022; Zhou et al., 2018), it could be highly valuable to further explore the interaction between situational and dispositional variables known to be associated with placebo responsiveness in future OLP studies. Lastly, examining additional moderators such as stress resilience and coping styles, together with subjective and physiological outcomes of OLP interventions, could help to obtain a more comprehensive understanding of observed OLP effects.

To conclude, this study extends previous evidence demonstrating OLP effects on both subjective and physiological stress responses in a real‐life stress scenario. Individuals with low dispositional optimism exhibited the most pronounced OLP effects on HCC, independent of treatment expectation, whereas the OLP effect on psychological distress was influenced by the interaction of optimism and expectation, indicating that these factors differently shape psychological and physiological OLP responses. Overall, these findings suggest the potential of OLPs to alleviate the psychophysiological impact of prolonged stress exposure.

## CONFLICT OF INTEREST STATEMENT

The authors have no conflict of interest.

## ETHICS STATEMENT

The study project was approved by the ethics committee of MSB Medical School Berlin (dossier MSB‐2021/60).

## REGISTRATION

The study project was registered in the German Clinical Trials Register (DRKS00031423, https://drks.de/search/de/trial/DRKS00031423).

## Supporting information


**Figure S1.** Trajectories of self‐reported psychological distress measures by group during the OLP intervention from T1 to T5. The plot represents estimated marginal means adjusted for sex, age, job hours, semester, degree program, significant past stressors, and baseline. Error bars represent ± 1 standard error of the mean. Trajectories are based on the same dataset reported in Liedtke et al. (2026).
**Table S1** Associations between psychological distress at baseline and dispositional optimism.
**Table S2** Intercorrelations of psychological distress indicators (AUCi variables).
**Table S3** Fit indices for alternative confirmatory factor models of psychological distress.
**Table S4** Estimated means of hair cortisol concentrations and psychological distress by group and level of optimism.
**Table S5** Estimated means of hair cortisol concentrations by group and levels of optimism and expectation.
**Table S6** Estimated means of psychological distress by group and levels of optimism and expectation.

## Data Availability

The referenced dataset that supports the findings of this study is available on the Open Science Framework (OSF), https://osf.io/9whjx.
