# Supplementary material for: Dispositional optimism and open‐label placebo responses in hair cortisol concentrations and psychological distress—A randomized controlled trial
Source: Appl Psychol Health Well Being. 2026 Sep 9;18(5):e70217. doi: 10.1111/aphw.70217 (PMC13555520; doi:10.1111/aphw.70217)
Supplement: Supplementary file 1 [file APHW-18-0-s001.docx]

**Supplementary Material for Applied Psychology: Health and Well-Being**

**Dispositional optimism and open-label placebo responses in hair cortisol concentrations and psychological distress – a randomized controlled trial**

Carolin Liedtke^1,^*, Michael Schaefer^1,2,†^, and Sören Enge^1,2,†^

^1^Department of Psychology, MSB Medical School Berlin, Germany

^2^Institute of Neuroscience and Biopsychology for Clinical Application, MSB Medical School Berlin, Germany

*Correspondence concerning this article should be addressed to Carolin Liedtke MSB Medical School Berlin, Rüdesheimer Straße 50, 14197 Berlin, Germany. carolin.liedtke@medicalschool-berlin.de, +4917644271996

^†^ These authors contributed equally to this work and share last authorship.

**Table S1**

*Associations between psychological distress at baseline and dispositional optimism*

|  | Optimism  (LOT-R) | Negative affect  (NA) | Test Anxiety  (PAF) | Subjective stress (DASS-S) | Exam-related stress (VAS) |
| --- | --- | --- | --- | --- | --- |
| Negative affect (NA) | -.467*** | - |  |  |  |
| Test Anxiety (PAF) | -.492*** | .468*** | - |  |  |
| Subjective stress (DASS-S) | -.441*** | .488*** | .518*** | - |  |
| Exam-related stress (VAS) | -.271*** | .275*** | .433*** | .444*** | - |
| Psychological distress (latent factor) | -.555*** | .705*** | .832*** | .843*** | .645*** |

*Note*. Partial correlation coefficients controlled for sex and age. Tests were two-sided. *** *p* < .001.

**Methods S1**

*Latent factor modeling of psychological distress at baseline*

To account for potential baseline differences, baseline psychological distress was modeled as a latent factor indicated by standardized baseline scores of negative affect (NA), test anxiety (PAF), subjective stress (DASS-S), and exam-related stress (VAS). The measurement model showed acceptable overall fit, χ²(2) = 6.61, *p* = .037, robust CFI = .976, robust TLI = .929, SRMR = .028. Although the chi-square test was significant and RMSEA was elevated (.107, 90% CI [.023, .202]), these indices should be interpreted cautiously given the very low degrees of freedom of the model. All indicators loaded significantly on the latent factor (λ = .58–.74, all *p* < .001), supporting the use of a common latent baseline distress construct as a covariate to control for baseline distress in the moderation analyses.

**Table S2**

*Intercorrelations of psychological distress indicators (AUCi variables)*

|  | Negative affect AUCi | Subjective stress AUCi | Test anxiety  AUCi |
| --- | --- | --- | --- |
| Subjective stress AUCi | .454*** | - |  |
| Test anxiety AUCi | .444*** | .276*** | - |
| Exam-related stress AUCi | .433*** | .306*** | .269*** |

*Note*. Pearson correlation coefficients are reported. Tests were two-sided. *** *p* < .001.

**Table S3**

*Fit indices for alternative confirmatory factor models of psychological distress*

| Model | *χ*²(df) | *p* | CFI | TLI | RMSEA [90% CI] | SRMR | AIC | BIC |
| --- | --- | --- | --- | --- | --- | --- | --- | --- |
| CFA 1 | .222 (2) | .895 | 1.000 | 1.047 | .000 [.000, .068] | .007 | 2162.712 | 2202.352 |
| CFA 2 | .007 (1) | .934 | 1.000 | 1.045 | .000 [.000, .052] | .001 | 2164.459 | 2207.402 |
| CFA 3 | .000 (0) | NA | 1.000 | 1.000 | NA | .000 | 1682.169 | 1711.899 |

*Note*. CFA = confirmatory factor analysis; NA = not applicable. All models were estimated using robust maximum likelihood estimation. CFA 1 reflects the one-factor model used in the main analyses (4 indicators: negative affect, test anxiety, subjective stress, exam-related stress). CFA 2 is an alternative two-factor model separating affective indicator (negative affect, test anxiety) from stress-specific indicators (subjective stress, exam-related stress). The latent correlation in the two-factor model was *r* = .94, indicating a lack of discriminant validity between factors. CFA 3 is a one-factor model with 3 indicators (test anxiety, subjective stress, exam-related stress), which was just-identified (df = 0). Alternative 3-indicator models revealed similar results. A fourth CFA with a two-factor model of general distress (negative affect, subjective stress) and exam-specific distress (test anxiety, exam-related stress) yielded an inadmissible solution, with a latent factor correlation of *r* = 1.02.

**Figure S1.** Trajectories of self-reported psychological distress measures by group during the OLP intervention from T1 to T5. The plot represents estimated marginal means adjusted for sex, age, job hours, semester, degree program, significant past stressors, and baseline. Error bars represent ± 1 standard error of the mean. Trajectories are based on the same dataset reported in Liedtke et al. (2026).

**Table S4**

*Estimated means of hair cortisol concentrations and psychological distress by group and level of optimism*

| Optimism | Hair cortisol | | Psychological distress | |
| --- | --- | --- | --- | --- |
|  | OLP group | Control group | OLP group | Control group |
| - 1 SD | -.072 | .070 | -.099 | .249 |
| Mean | -.051 | .022 | -.186 | .180 |
| + 1 SD | -.029 | -.026 | -.272 | .110 |

*Note.* Values are derived from the moderated regression model including the interaction of group and optimism.

**Table S5**

*Estimated means of hair cortisol concentrations by group and levels of optimism and expectation*

| Optimism | Hair cortisol | | | | | |
| --- | --- | --- | --- | --- | --- | --- |
|  | OLP group | Control group | OLP group | Control group | OLP group | Control group |
| - 1 SD | -.078 | .093 | -.074 | .057 | -.070 | .022 |
| Mean | -.051 | .040 | -.051 | .019 | -.051 | -.002 |
| + 1 SD | -.025 | -.013 | -.028 | -.019 | -.032 | -.025 |
| Expectation | - 1 SD | | Mean | | + 1 SD | |

*Note.* Values are derived from the moderated regression model including the three-way interaction of group, optimism, and expectation.

**Table S6**

*Estimated means of psychological distress by group and levels of optimism and expectation*

| Optimism | Psychological distress | | | | | |
| --- | --- | --- | --- | --- | --- | --- |
|  | OLP group | Control group | OLP group | Control group | OLP group | Control group |
| - 1 SD | -.126 | .444 | -.127 | .206 | -.128 | -.032 |
| Mean | -.093 | .225 | -.187 | .163 | -.281 | .101 |
| + 1 SD | -.061 | .005 | -.248 | .120 | -.435 | .235 |
| Expectation | - 1 SD | | Mean | | + 1 SD | |

*Note.* Values are derived from the moderated regression model including the three-way interaction of group, optimism, and expectation.

**References**

Liedtke, C., Schaefer, M., & Enge, S. (2026). Open-label placebo effects on psychological distress and hair cortisol in a randomized controlled trial – the moderating role of the five-factor personality traits. *Comprehensive Psychoneuroendocrinology*, *27*, 1–10. https://doi.org/https://doi.org/10.1016/j.cpnec.2026.100361
